# Hijacking of Lipid Droplets by Hepatitis C, Dengue and Zika Viruses—From Viral Protein Moonlighting to Extracellular Release

**DOI:** 10.3390/ijms21217901

**Published:** 2020-10-24

**Authors:** Alexandra P.M. Cloherty, Andrea D. Olmstead, Carla M.S. Ribeiro, François Jean

**Affiliations:** 1 Amsterdam UMC, Amsterdam Institute for Infection & Immunity, Department of Experimental Immunology, University of Amsterdam, Meibergdreef 9, 1105 AZ Amsterdam, The Netherlands; a.p.cloherty@amsterdamumc.nl (A.P.M.C.); c.m.ribeiro@amsterdamumc.nl (C.M.S.R.); 2 Department of Microbiology and Immunology, Life Sciences Institute, University of British Columbia, 3559–2350 Health Sciences Mall, Vancouver, BC V6T1Z3, Canada; aolmstea@mail.ubc.ca

**Keywords:** lipid droplets, SREBP (sterol regulatory element-binding protein) pathway, autophagy, hepatitis C virus, dengue virus, Zika virus

## Abstract

Hijacking and manipulation of host cell biosynthetic pathways by human enveloped viruses are essential for the viral lifecycle. *Flaviviridae* members, including hepatitis C, dengue and Zika viruses, extensively manipulate host lipid metabolism, underlining the importance of lipid droplets (LDs) in viral infection. LDs are dynamic cytoplasmic organelles that can act as sequestration platforms for a unique subset of host and viral proteins. Transient recruitment and mobilization of proteins to LDs during viral infection impacts host-cell biological properties, LD functionality and canonical protein functions. Notably, recent studies identified LDs in the nucleus and also identified that LDs are transported extracellularly via an autophagy-mediated mechanism, indicating a novel role for autophagy in *Flaviviridae* infections. These developments underline an unsuspected diversity and localization of LDs and potential moonlighting functions of LD-associated proteins during infection. This review summarizes recent breakthroughs concerning the LD hijacking activities of hepatitis C, dengue and Zika viruses and potential roles of cytoplasmic, nuclear and extracellular LD-associated viral proteins during infection.

## 1. Introduction

**Figure 1:**
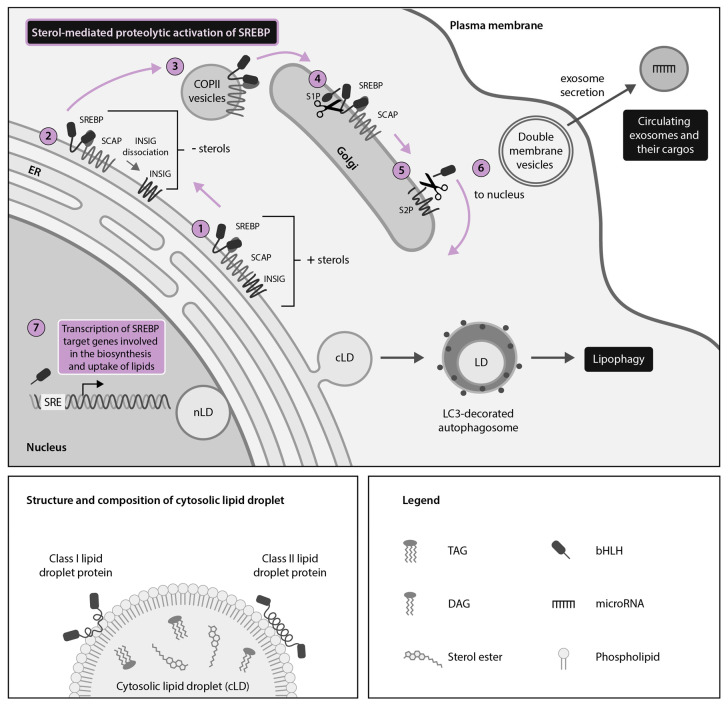
The sterol regulatory element-binding protein (SREBP) pathway is a master regulator of cellular lipid droplet (LD) metabolism. LDs are spherical organelles composed of a phospholipid monolayer surrounding a core of neutral lipids. LDs have been described in both the cytoplasm (cLDs) and nucleus (nLDs) of homeostatic cells, with cLDs being better described. cLDs serve as docking platforms for Class I and Class II host LD proteins. LD metabolism is controlled by endoplasmic reticulum (ER)- and nuclear membrane-bound proteins called SREBPs, which control expression of key target genes involved in the biosynthesis and uptake of cholesterol and other lipids. When sterol levels are high, SREBPs are retained in an inactive state in the ER by SREBP-cleavage activating protein (SCAP) and insulin-induced gene (INSIG) (**1**). When sterol levels are low, INSIG dissociates from SCAP at the ER (**2**) and coat protein II (COPII)-coated vesicles transport SREBP-SCAP complexes to the Golgi apparatus (**3**), where the active N-terminal portion of SREBPs is cleaved by site-1 protease (S1P) (**4**) and site-2 protease (S2P) (**5**). The N-terminal portion of SREBPs then translocates to the nucleus (**6**) to act as a transcription factor coordinating expression of many lipid-metabolism related genes (**7**) that can subsequently impact LD biogenesis. SREBP regulation of LD metabolism can be further fine-tuned by exosome-packaged host microRNAs (miR) such as miR-29 (inhibits SCAP and SREBP-1 expression), miR-33 (inhibits SREBP-1 expression) and miR-24 (inhibits INSIG expression). SREBP-regulated LD homeostasis also intersects with other cellular pathways such as lipophagy, which catabolizes LDs in microtubule-associated protein light chain 3 (LC3)-decorated autophagosomes to liberate their component lipids.

Lipid droplets (LDs) are highly conserved intracellular organelles that were long conceptualized as merely storage sites for neutral lipids; however, they have recently been implicated in a variety of novel functions. The surface of LDs is composed of a phospholipid monolayer with a unique fatty acid composition as compared to other intracellular organelles (Figure 1) [1]. This monolayer surrounds a core of hydrophobic neutral lipids, including triacylglycerol and cholesterol esters [2]. LDs are predominantly located in the cytoplasm (cLDs) but they have also been reported to associate with a variety of membranous organelles, including the endoplasmic reticulum (ER) where cLDs are derived; more recently, they have been found in the nucleus (nLDs) (Figure 1) [3,4]. Furthermore, extracellular LDs (eLDs) were recently described as being released by virally infected human cells, where they are may play an important role in the extracellular milieu in regards to viral pathogenesis [5].

LDs are now recognized as dynamic organelles with functionality in fatty acid trafficking, lipid signaling and host defense against intracellular pathogens such as viruses [6,7]. Despite their potential defensive roles, viruses of the *Flaviviridae* virus family extensively manipulate LDs for viral replication and production [8,9]. Members of the *Flaviviridae* family comprise a large group of enveloped viruses with positive-sense single-stranded RNA genomes. The *Flaviviridae* family includes many medically important viruses including hepatitis C virus (HCV), a member of the *Hepacivirus* genus and several members of the *Flavivirus* genus, including dengue virus (DENV), Zika virus (ZIKV) and Japanese encephalitis virus (JEV), among others. Members of the *Pegivirus* and *Pestivirus* genera also belong to the *Flaviviridae* family. The pegiviruses include GB virus C (formerly called hepatitis G virus), a common non-pathogenic human virus [10], while pestiviruses, such as bovine viral diarrhea virus (BVDV), are important animal pathogens [11].

Hepaciviruses and flaviviruses encode a capsid protein, two envelope proteins and several non-structural (NS) proteins (Table 1). The structure and charge of the viral capsid, which localizes to cLDs, is relatively conserved [12,13,14,15]. *Flaviviridae* enter host cells via receptor-mediated endocytosis and rely on acidification of endosomes by fusion with lysosomes to trigger viral envelope fusion and subsequent uncoating of the genome [16,17]. Replication of the virus occurs within highly organized cytoplasmic viral replication factories, with the viral genome acting as mRNA for direct translation of the viral polyprotein [18]. Both the endoplasmic reticulum (ER) and LDs are used as assembly platforms for progeny virions, after which viruses bud into the ER lumen and mature as they are transported through the host canonical ER-Golgi secretory pathway towards the cell surface for viral release [19,20].

HCV remains the best studied and best understood of the *Flaviviridae* family in terms of its interaction with LDs. HCV hijacks a portion of the hepatic very low density lipoprotein (VLDL) assembly pathway to form so-called lipoviroparticles (LVPs) or low-density infectious viral particles; these are rich in cholesterol and triglycerides and contain apolipoproteins [21,22]. Assembly of HCV particles is initiated at the surface of LDs where several HCV proteins co-localize. LDs are also likely the main source of triglycerides exported into HCV LVPs, as they are a major source of triglycerides for VLDLs [23]. In light of recent epidemics caused by flaviviruses, there is also increasing evidence that DENV, ZIKV, JEV and others extensively use LDs during infection to hijack host lipid metabolism for energy and to promote viral replication and assembly [5,7,9,13,24,25]. A study on BVDV suggests that the interaction with host LDs may also be conserved in pestivirus infections [26].

In this review, we summarize recent breakthroughs concerning viral hijacking of LDs by HCV, DENV and ZIKV with a focus on the sterol regulatory element-binding protein (SREBP) pathway and autophagy. We also discuss the roles that LD-associated viral proteins play during infection and explore the potential roles of the recently described nLDs and eLDs in viral protein sequestration and protein homeostasis during infection. An improved understanding of viral manipulation of LD metabolism and interaction of LDs with intra- and extracellular compartments and signaling pathways may well set the stage for improved host-directed therapeutics against *Flaviviridae* [27].

## 2. HCV, DENV and ZIKV Hijack Cytoplasmic Lipid Droplets (cLDs) Through Dysregulation of the SREBP Pathway

The SREBP pathway is a master regulator of LD homeostasis (Figure 1). Specifically, the SREBP pathway is key for host regulation of cholesterol and lipid homeostasis as well as for the HCV, DENV and ZIKV lifecycle [28,29,30,31]. SREBPs are ER- and nuclear membrane-bound transcription factors that control the expression of key lipogenic enzymes, thereby integrating multiple cellular signals for the control of lipogenesis and subsequent LD biogenesis [28,32]. There are three mammalian SREBP isoforms: SREBP-1a, SREBP-1c and SREBP-2, which respectively control fatty acid and cholesterol synthesis, fatty acid synthesis and cholesterol synthesis and uptake [28,33].

For the N-terminal portion of SREBPs to enter the nucleus, the SREBPs must be translocated to the Golgi body by the SREBP-cleavage activating protein (SCAP) and subsequently cleaved in a two-step proteolytic cascade involving the human subtilisin kexin isozyme-1/site-1 protease (SKI-1/S1P) and the site 2 protease (S2P) (Figure 1) [32,34,35,36].

SCAP-mediated translocation of SREBPs to the Golgi body requires dissociation of SCAP from the regulatory protein insulin-induced gene (INSIG) at the ER and it occurs when sterol levels are low [36]. Once in the nucleus, cleaved SREBPs regulate the expression of genes such as proprotein convertase subtilisin kexin 9 (PCSK9), which encodes a protein that binds the low-density lipoprotein receptor (LDLR) and targets it for lysosomal degradation (Figure 1) [37].

Host microRNAs (miRNAs) are conserved short non-coding RNAs that regulate host gene expression with implications in human lipid metabolism and the innate immune system. Several human miRNAs have been implicated in the regulation of the SREBP pathway [38,39] (Figure 1). For example, miRNA (miR)-29 and miR-33 target the first steps of the SREBP pathway by preventing expression of SCAP and SREBP-1 to downregulate fatty acid synthesis and LD biogenesis [40,41] (Figure 1). Downstream in the SREBP pathway, miR-24 inhibits INSIG and thereby promotes SREBP activation and lipogenesis [42]. Regulation of lipid metabolism by miRNAs may have a far-reaching intercellular impact, for example, via miRNA packaging in extracellular vesicles termed exosomes or in association with lipoproteins, which are also demonstrated carriers of host miRNAs, with implications for intercellular communication, immunity and lipid metabolism [39,43] (Figure 1). Thus, miRNAs also represent a regulatory mechanism of lipid metabolism with great potential for therapeutic antiviral manipulation [31].

### 2.1. HCV Usurps the Host SREBP Pathway Through Multiple Mechanisms to Permit the Robust Replication and Production of Infectious Viral Particles

**Figure 2:**
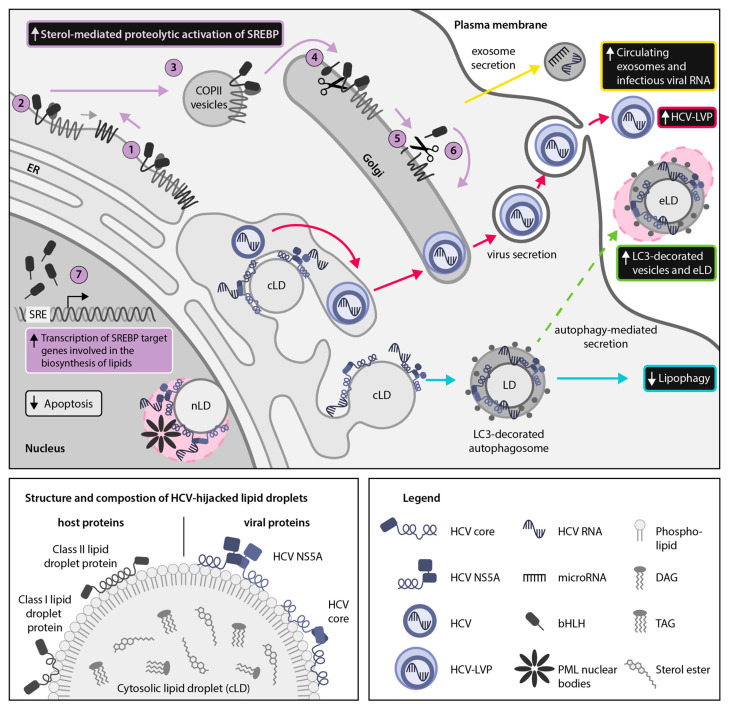
Hepatitis C virus (HCV) hijacks sterol regulatory element-binding protein (SREBP)-regulated host lipid metabolism. HCV relies on manipulation of host lipid metabolism for virion packaging and release as lipid-coated lipoviroparticles (LVPs) (red arrows). Thus, HCV infection results in a multi-faceted disruption of lipid homeostasis, including remodeling of intracellular lipids into a membranous web, increased sterol-mediated SREBP activation and transcription of SREBP target genes (purple arrows: (**1**–**7**)), reduced autophagic catabolism of lipid droplets (LDs) (cyan arrows) and enhanced secretion of exosomes containing infectious viral RNA and host microRNAs that regulate lipid metabolism (yellow arrows). Here, we propose that the cytoplasmic LD (cLD)-associated HCV proteins core and non-structural protein 5A (NS5A) may also use nuclear LDs (nLDs) as a sequestration platform for interfering with host LD-associated proteins (pink circle). In the nucleus, LD-association of NS5A and core may facilitate their interaction with promyelocytic leukemia (PML) nuclear bodies, resulting in inhibition of host cell apoptosis. In addition, we hypothesize that hijacking of secreted microtubule-associated protein light chain 3 (LC3)-decorated vesicles containing LDs may be an additional mechanism for release of HCV virions, viral proteins or infectious viral RNA to escape the host cell (green arrow). Hypothetical pathways are highlighted with dotted lines.

Several studies have confirmed the importance of SREBP pathway activation during HCV infection (Figure 2). Transient increases in the level of expression and the proteolytic activation of both SREBP-1 and SREBP-2 have been found during HCV infection, followed by increased lipogenesis of cholesterol and membrane lipids [8,19,44]. Specifically, the HCV core, NS4B, NS5A and the 3′-untranslated region (UTR) have been found to activate SREBP signaling pathways, thereby enhancing the activity of a variety of enzymes involved in lipid biosynthesis (Table 2) [44,45,46,47,48,49,50,51].

Activation of SREBP signaling and of lipid biosynthesis has been demonstrated as occurring through several mechanisms. One such mechanism of HCV-induced activation of SREBP occurs through oxidative stress-associated pathways [44,46]. HCV infection, HCV NS4B and core induce oxidative stress, resulting in activation of the phosphatidylinositol 3-kinase (PI3K) / protein kinase B (AKT) pathway. PI3K-AKT activation results in increased phosphorylation and transactivation of SREBPs [44].

Recently, HCV NS5A was found to trigger accumulation of hepatic LDs in a mouse model through a 5′ adenosine monophosphate-activated protein kinase (AMPK)-SREBP-1c-dependent pathway [45]. AMPK is a master sensor of cellular energy levels and plays an important role in regulating lipid metabolism, including fatty acid synthesis and oxidation. NS5A was shown to inhibit AMPK activation, which subsequently resulted in increased expression of SREBP-1c and other lipid metabolism genes along with increased LD levels [45].

The DEAD-box helicase 3 X-linked (DDX3X)-IκB kinase α (IKKα) axis has also been implicated in HCV-mediated SREBP activation [50]. DDX3X is a proviral host factor that recognizes the HCV 3′-UTR, thereby triggering IKKα activation and subsequent induction of LD biogenesis and viral assembly. IKKα is a regulator of the NFκB pathway but upon DDX3X-dependent activation, it promotes SREBP expression and induction in an NFκB-independent manner. While IKKα translocates to the nucleus to promote lipogenesis, DDX3X remains in the cytoplasm and subsequently co-localizes with LDs and HCV core and nonstructural proteins [51]. This DDX3X-IKKα-SREBP axis is crucial for productive HCV infection, underscoring that HCV hijacking of the SREBP pathway is multifaceted [50,51]. The overall importance of the SREBP pathway and LD formation is further solidified by studies demonstrating that by blocking SKI-1/S1P, a major regulator of SREBPs, LD formation can be restricted and HCV infection blocked in vitro [29,54] (Figure 2).

Interestingly, epidemiological studies have shown that patients with long-term, chronic HCV infections have lower circulating lipid levels, namely decreased cholesterol and LDL levels, than patients who achieve a sustained virologic response (SVR) [55]. This may be due to rerouting of lipids to create a membranous web and to assemble viral particles on LDs, thereby reducing circulating lipids, underscoring the extensive lipid remodeling that occurs during HCV infection. Correspondingly, it was recently reported that concentrations of the SREBP target gene, PCSK9, which normally correlates with decreased LDLR in the liver but heightened concentration of LDL in plasma, were significantly increased *in vivo* in patients who achieved SVR as compared to non-responders [31]. Interestingly, PCSK9 has also been shown to inhibit HCV infection in vitro, perhaps by downregulating LDLR, which functions as an HCV receptor [31,56,57,58]. Altogether, this indicates differential remodeling of intra-and extracellular lipid concentrations by HCV, with the SREBP pathway playing a key role.

Further highlighting the central role of the SREBP pathway in HCV infection, recent studies have identified that concentrations of the lipoprotein- and exosome-associated miR-24 and miR-223, which enhance SREBP processing and cholesterol efflux, respectively, were increased in HCV sustained virologic responders as compared to non-responders [31,42,52]. This may reflect a recovery of normal lipid metabolism, permitting upregulation of extracellular lipoproteins that carry these miRNAs and it further suggests a complex interplay between exosome-associated miRNAs and the SREBP pathway during HCV infection (Figure 2). Considering that exosome-associated miRNAs are rapidly becoming important biopharmaceuticals for treating a variety of conditions [59,60], improved understanding of the role of miRNAs in regulating the SREBP pathway could open up new possibilities for countering viral hijacking of lipid metabolism.

### 2.2. DENV and ZIKV Divert the SREBP Pathway to Support Their Lifecycle and the Induction of Host Antiviral Responses

**Figure 3:**
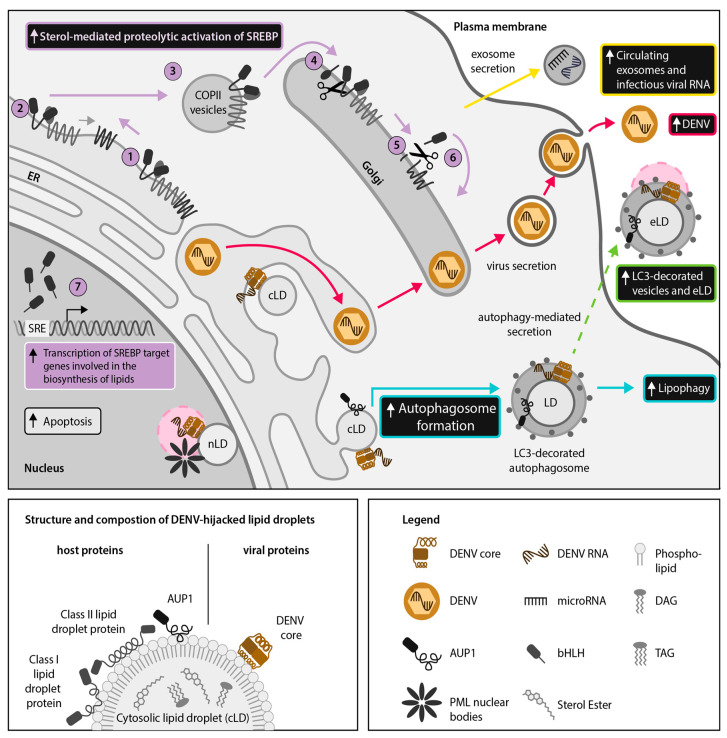
Dengue virus (DENV) dysregulates host lipid metabolism. DENV perturbs host lipid homeostasis by enhancing sterol regulatory element-binding protein (SREBP) activation (purple arrows: (**1**–**7**)), leading to increased lipid droplet (LD) formation. DENV stimulates LD degradation via lipophagy by enhancing (ancient ubiquitous protein 1) AUP1 translocation from cytoplasmic LDs (cLDs) to microtubule-associated protein light chain 3 (LC3)-decorated autophagosomes (cyan arrows). Enhanced LD catabolism provides free fatty acids to serve as an energy source for production of infectious virions (red arrows). A recent report has shown that infectious viral RNA and extracellular LDs (eLDs) are contained within LC3-decorated extracellular vesicles in DENV-infected cells, suggesting that eLDs could be used as anchors for viral proteins. We hypothesize that the cLD-associated DENV core protein may also co-localize with eLDs in order to facilitate extracellular dissemination of viral RNA (green arrows). In addition, we propose that the DENV core protein may utilize nLDs as a scaffold for interacting with promyelocytic leukemia (PML) nuclear bodies (pink circle), thereby promoting host cell apoptosis and suppressing host interferon responses. Hypothetical pathways are highlighted with dotted lines.

It was recently shown that DENV hijacks SKI-1/S1P-mediated activation of the SREBP pathway [30] (Figure 3). Similar to SKI-1/S1P inhibition blocking HCV infection as described above [29], treatment of Huh-7.5.1 cells with the SKI-1/S1P active site-directed inhibitor PF-429242 resulted in dose-dependent restriction of DENV infection and could be reversed by treatment with oleic acid, an inducer of LD biogenesis [30]. Recent parallel experiments with ZIKV have also demonstrated a dose-dependent reduction in infection using PF-429242 (unpublished data; Jean et al.). Similarly, a synthetic agonist with SREBP-binding activity, AM580, exhibited antiviral activity against ZIKV infection [61].

Recent publications suggest cross-talk between the SREBP pathway and the host interferon response during flavivirus infection. SCAP, which is required for SREBP cleavage and activation (Figure 1), prevents ubiquitination of DENV protein NS3 and thereby inhibits the DENV NS2B-NS3 serine-protease heterocomplex from cleaving host antiviral protein stimulator of interferon genes (STING) [53]. Ectopic expression of SCAP, which was hypothesized to enhance STING functioning by inhibiting NS2B-NS3, resulted in impaired DENV infection, while knockdown of SCAP facilitated DENV infection [53]. This suggests that the SREBP pathway is involved in inducing the host antiviral response in reaction to viral disruption of LD homeostasis during DENV infection and may play a similar role in ZIKV infection.

## 3. Discovery of Nuclear LDs (nLDs) and Emerging Moonlighting Activities of LD-Associated Viral Proteins in the Host-Cell Nucleus of Cells Infected With HCV, DENV and ZIKV

Traditionally, LDs were thought to exist only in the cytoplasm of eukaryotic cells. While cLDs originate from the ER, nLDs originate directly from the inner nuclear membrane (INM) [3,4]. nLDs remain associated with the INM via the predicted transmembrane protein Seipin, which also plays a pivotal role in cLD maturation at the ER in complex with LD assembly factor 1 (LDAF1) [3,62]. Because nLDs have only been recently recognized, their roles have not been fully characterized but it is logical to hypothesize that much like cLDs, they may act as suppliers of lipids for membrane expansion and as scaffolding platforms for proteins [63,64]. Notably, nLDs are also associated with promyelocytic leukemia (PML) nuclear bodies, which are punctate structures within the nuclear matrix implicated in regulation of transcription and apoptosis in response to a variety of stresses, including during antiviral defense [65,66] (Figure 2 and Figure 3).

Although *Flaviviridae* replicate in the cytoplasm, there have been many reports of viral proteins, including the HCV, DENV and JEV capsid proteins, localizing to the nucleus [24,67,68,69]. In light of the recent discovery of nLDs, it is time to revisit these observations. We are the first to propose that localization of HCV proteins to LDs in alternative subcellular compartments could potentiate ‘moonlighting’ or the assumption of additional functions by viral proteins depending on their localization and interacting partners [70]. We suggest that targeting of *Flaviviridae* proteins to nLDs could facilitate currently understudied viral hijacking mechanisms (Figure 2 and Figure 3). Localization of viral proteins to nLDs could increase protein stability and half-life within the nucleus, potentially permitting prolonged viral manipulation of host metabolic pathways as discussed below.

Since the early 2000s, there have been several reports of HCV and flavivirus core proteins localized to the host nucleus [13,14,67,68]. Core proteins are LD-associated, bind RNA and include a conserved predicted nuclear localization signal (NLS) [14,22]. Interestingly, both HCV and DENV core proteins have been demonstrated to interact with PML nuclear bodies [67,71] (Figure 2 and Figure 3). During DENV infection, nuclear localization of the C protein is required for its interaction with the PML nuclear body associated-host protein, death domain associated protein (DAXX), to promote Fas-mediated apoptosis in HepG2 cells, which may contribute to viral release or dengue hemorrhagic fever pathophysiology (Figure 3) [67].

While DENV promotes apoptosis via interaction with PML nuclear bodies, the HCV core protein inhibits PML isoform IV-induced apoptosis (Figure 2) [71]. This core-mediated inactivation of the PML tumor suppressor pathway, which involves regulation of the key nuclear tumor suppression protein p53, may promote HCV-associated hepatocarcinogenesis [72].

Furthermore, the HCV LD-associated protein NS5A also includes a C-terminal NLS and has been found in membrane fractions corresponding to the nuclear periplasmic membrane and in association with p53 [69,73]. HCV NS5A exists in multiple phospho-isoforms with no known enzymatic function but it is an essential component of HCV replication and exerts a wide range of effects on cellular pathways and processes, including innate immunity and host cell growth and proliferation [74]. HCV NS5A inhibits apoptosis of host cells via p53-mediated transcriptional modulation of the apoptosis-related p21 gene and by inhibiting Bcl-2 associated X protein (Bax)-induction of apoptosis in a manner analogous to host regulatory protein Bcl-2 [70,75]. Additionally, HCV NS5A interferes with the host interferon response by interacting with the host kinase rapidly accelerated kinase 1 (Raf1), a member of the Raf/Ras/MEK pathway, which regulates expression of interferon-stimulated genes and interferon receptors, subsequently resulting in increased viral replication [76]. A naturally occurring caspase-cleaved form of HCV NS5A has been shown to translocate into the nucleus in association with Raf1, interfering with HCV replication [77].

Recent reports indicate that this functionality may be conserved among flaviviruses [78,79]. The flavivirus NS5 protein, which consists of an N-terminal methytransferase and a C-terminal RNA-dependent RNA polymerase, also contains NLSs and localizes to the host nucleus. Nuclear localized ZIKV NS5, for example, has been demonstrated to suppress activation of type I interferon transcription and downstream responses [78,79].

Based on the described findings above, we suggest that localization of *Flaviviridae* core and NS5 proteins (HCV NS5A; DENV/ZIKV NS5) to nLDs could represent a mechanism for sustained interaction with host nuclear proteins, leading to either efficient induction or suppression of host cell apoptosis, promoting viral release or contributing to virus-associated carcinogenesis, respectively, as well as interfering with host interferon responses [67,70,71,72,75,76,78,79]. We propose that future studies of nuclear localized *Flaviviridae* proteins should examine their interactions with nLDs, as therapeutically targeting nLDs may represent a strategy to counteract such virus manipulation of host apoptotic and interferon signaling.

## 4. DENV, ZIKV and HCV Hijack Secretory Autophagy for Viral Dissemination and Release of Extracellular LDs (eLDs) During Infection

It is well established that *Flaviviridae* members manipulate autophagy, a process best known for its degradative function [9,80,81]. During degradative autophagy, intracellular cargo is enveloped by double-membrane microtubule associated protein 1 light chain 3 (LC3)-II-positive vesicles known as autophagosomes. (Figure 2 and Figure 3) Autophagosomes may subsequently fuse with lysosomes resulting in the degradation of enveloped contents. Alternatively, it has been suggested that HCV may hijack autophagy proteins to promote translation of incoming HCV RNA and initiate viral replication [82]. The role of autophagy in regulating host lipid homeostasis and innate immune responses, including the interferon response, may also be targeted by HCV [45,80].

In contrast, the leading theory regarding the role of autophagy in the pathogenesis of DENV and ZIKV has centered around lipophagy, or the autophagic degradation of LDs [9,83]. Lipophagy is responsible for the release of free fatty acids for the purposes of energy mobilization, quality control of LD-targeted proteins and lipid homeostasis [84]. During DENV or ZIKV infection, initial upregulation of LD biogenesis is followed by lipophagy induction, resulting in increased release of fatty acids that undergo β-oxidation in the mitochondria, thereby liberating energy for viral replication and assembly (Figure 3) [9,83].

Autophagy also plays an important role in the secretion of intracellular material, such as IL-1β and ferritin [85,86]. The mechanism of secretory autophagy, particularly in the context of viral infection, is not completely understood (Figure 3). However, it has been established that host proteins such as the pro-inflammatory cytokine interleukin-1β (IL-1β) are sequestered into LC3-II-positive autophagosomal membranes by a system of proteins including tripartite motif-containing 16 (TRIM16) and R-SNAP receptor (SNARE) Sec22B [86,87]. Plasma membrane syntaxins and synaptosomal-associated proteins are later required for fusion of the cytoplasmic autophagosome with the plasma membrane, leading to cargo release [86].

The structure of extracellular secretory autophagosomes has not been fully elucidated but secretory autophagosomes have been confirmed to represent a subset of exosomes isolated by ultracentrifugation [88]. Notably, a recent report identified LDs contained within secretory autophagosomes released from DENV-infected cells, suggesting that this may be a mechanism for extracellular transport of cLDs [5] (Figure 3). Although this phenomenon has not been fully studied, extracellular transport of LDs may potentiate moonlighting of LD-associated host and viral proteins in the extracellular milieu. These novel findings suggest that the intersection among the exosomal secretory pathway, LDs and autophagy in *Flaviviridae* infections warrants further examination (Figure 3).

### 4.1. DENV and ZIKV Use Autophagy for Extracellular Transport of LDs and LD-Associated Proteins

For several years, the prevailing model regarding the role of autophagy during DENV and ZIKV infections has been that the viruses upregulate lipophagy to release free fatty acids from LDs, for subsequent catabolic processing via β-oxidation [9]. β-oxidation generates ATP, which is necessary for efficient replication of DENV [83]. In alignment with this model, LD volume is decreased during DENV infection as measured by electron microscopy, and treatment of Huh-7.5.1 cells with exogenous fatty acids was able to complement defects in DENV replication after inhibition of autophagy [83]. In line with this, recent data further suggest that smaller LDs are preferentially targeted for autophagy rather than lipolysis [89].

It was recently established that the DENV NS4A and NS4B proteins bind and translocate ancient ubiquitous protein 1 (AUP1) from LDs to autophagosomes, resulting in upregulation of lipophagy (Figure 3) [9]. AUP1 normally resides on LDs and the ER and it has previously been implicated in both LD accumulation and ER protein quality control but it is not a general requirement for autophagy induction [90]. This suggests that AUP1 may be a lipophagy-specific factor. This would explain the specific upregulation of lipophagy by DENV and ZIKV, while other forms of autophagy, including autophagic degradation of the ER, are downregulated by flaviviruses [91].

The mechanism of lipophagy triggering by AUP1 has not been fully elucidated but it requires deubiquitinylation of AUP1 by DENV and stimulation of AUP1 acyltransferase activity by DENV NS4A and NS4B [9]. Furthermore, knockout of AUP1 by clustered regularly interspaced short palindromic repeats (CRISPR)-Cas9 genome editing technology in the human HepG2 cell line eliminated production of infectious DENV and ZIKV progeny [9]. Likewise, ZIKV NS4A and NS4B proteins have been found to induce lipophagy in human fetal neural stem cells, which results in inhibition of neurogenesis [81].

Notably, a recent study found that DENV antigens, infectious DENV RNA and LDs were present in secretory autophagosomes released by human Huh-7 cells lines, indicating, for the first time, secretory autophagy as a potential mechanism for extracellular transport of LDs (Figure 3) [5]. Furthermore, there are indications that ZIKV may use secretory autophagy during transmission through the placental barrier from mother to child but it is not yet known if ZIKV-associated secretory autophagosomes also carry eLDs [92]. A recent study also found that ZIKV induces upregulation of LD size and abundance in uninfected neighboring placental cells [25]. It would be interesting to investigate the potential involvement of secretory autophagy in this bystander mechanism. Considered together, these findings suggest that DENV and ZIKV may initially upregulate lipophagy to generate ATP but subsequently in the viral lifecycle, they may specifically upregulate and hijack secretory autophagy for further manipulation of lipid metabolism and viral dissemination.

### 4.2. HCV Employs the Secretory Autophagy Pathway for Viral Dissemination

Exosomes have a well-established role during HCV infection [93]. For example, patient-derived exosomes have been demonstrated to transmit infectious HCV RNA associated with host miR-122, argonaute 2 (Ago2) and heat shock protein (HSP) 90 (Figure 2), all of which enhance HCV replication [94]. Notably, the knock-down of autophagy protein Beclin1, which is important for autophagosome assembly, has been shown to inhibit release of infectious HCV particles via the exosomal pathway in Huh-7 cells [95]. This indicates that infectious HCV particles could be released via autophagy-associated secretion mechanisms, which likely intersect with the exosomal pathway. HCV has additionally been found to upregulate autophagy at earlier points in its lifecycle. For example, translation of incoming HCV RNA in Huh-7 cells was reported to depend on virus-enhanced conversion of cytosolic LC3-I to its autophagosome membrane-bound form LC3-II [82].

Considering the critical connection between autophagy and exosomes during HCV infection, combined with the recent finding that eLDs are transported via secretory autophagosomes released from a DENV-infected cell line [5], a pressing question remains whether LDs are also transported extracellularly during HCV infection. LDs contained within secretory autophagosomes have great potential for stabilizing associated host and viral proteins during transport and for subsequent delivery to bystander cells during viral infection (Figure 2). For example, delivery of viral components to the cytoplasm could trigger a pre-emptive interferon response in non-neighboring bystander cells [96]. It is also possible that secretory autophagosomes could carry lipoprotein- and exosome-associated miRs, such as miR-24, miR-122 and miR-223, during HCV infection [94]. Increased circulating levels of both miR-24 and miR-223 are associated with SVR in HCV-infected patients, which illustrates how miR-mediated intercellular communication could limit *Flaviviridae* pathogenesis [31,42,52]. It remains to be investigated whether extracellular LDs indeed promote early innate defenses to DENV [5] or other *Flaviviridae* or if such LDs are somehow hijacked for viral dissemination.

## 5. Discussion

In this review, we present the latest insights into the hijacking of LDs by *Flaviviridae* members HCV, DENV and ZIKV via manipulation of the SREBP pathway, a master regulator of LD biogenesis, as well as manipulation of the recently identified nLDs and eLD-containing secretory autophagosomes [3,5,30]. Based on these insights, we propose a model of LD hijacking by *Flaviviridae* across multiple cellular locations. Although the role of cLDs in HCV, DENV and ZIKV pathogenesis has been well reviewed, we believe that we are the first to highlight the potential roles of nLDs and eLDs as well as the important role of the SREBP pathway and exosome-associated miR during *Flaviviridae* infections [18,19,27]. Furthermore, we present a novel paradigm in viral hijacking of LDs, namely the potential moonlighting functions of LD-associated viral proteins arising as a result of their adventitious interactions with host factors in the nucleus, cytoplasm or extracellular milieu during the *Flaviviridae* lifecycle (Figure 2 and Figure 3). Future research should take into account the potential moonlighting capabilities of LD-associated viral proteins depending on their localization, and should identify whether LDs are also present within additional subsets of extracellular vesicles or other eukaryotic intracellular compartments. Notably, because LDs are conserved not only across eukaryotes but also in prokaryotes, the existence of nLDs suggests that LDs could also be present within mitochondria, which are also derived from endosymbionts [97,98]. It should be noted that although the role of LDs is well established in HCV and in several flaviviruses, further research is needed to establish the extent to which these findings apply to the lesser studied pestiviruses and pegiviruses. The finding that BVDV NS5A localizes to LDs [26] and that GB virus C particles are secreted as low density particles similar to HCV [99] suggests that LDs are likely to be important across the *Flaviviridae* family.

Development of much-needed antiviral treatments would benefit from an improved understanding of how viral proteins and virus-targeted therapeutics may exert different functionalities depending on the specific cellular location targeted. Adjusting existing LD model systems into three-dimensional (3D) organoid models could greatly facilitate such research, as it would permit more valid assessments of not only intra- but also extracellular communication [100]. Indeed, investigation into the role of LDs dependent on their localization could inform the development of host-targeting antivirals that could be broadly therapeutic against not only HCV, DENV, ZIKV and other understudied *Flaviviridae* members but also other lipid-hijacking pathogens from *Arenaviridae* and rotaviruses to the bacteria Chlamydia and Mycobacteria [7]. In light of the current coronavirus COVID-19 pandemic caused by the severe acute respiratory syndrome coronavirus 2 (SARS-CoV-2), a fundamental question also remains whether the pathways and antiviral targets discussed here have relevant similarities to pathogenic human coronaviruses. Notably, a recent report demonstrated that an inhibitor of SREBP activation has antiviral activity against SARS-CoV-1 and Middle East respiratory syndrome (MERS)-CoV [61]. Recent preliminary evidence also indicates that inhibitors of lipid metabolism and autophagy can suppress SARS-CoV-2 replication in vitro, [101]. Host-targeting antivirals have a clear advantage in that they are active across many genotypes and have a high genetic barrier to resistance development [27,102,103]. Specifically, host-directed RNA interference (RNAi)-based antivirals represent an intriguing novel mechanism that is gaining hold in the biopharmaceutical industry and are attractive for use in seasonal prophylaxis for endemic disease, such as the mosquito-transmitted DENV and ZIKV.

Finally, better understanding of LD functioning and malfunctioning could also shed light on therapeutic interventions for genetic lipid-associated diseases such as familial hypercholesterolemia and familial chylomicronemia and for promoting selective drug accumulation and activation within LDs [104]. Control of LD abundance by SKI-1/S1P inhibitors like PF-429242 or even diet, which can modify LD sizes and proteomes, may also prove to be an attractive option for control of *Flaviviridae* [30,105]. It has been reported that lifestyle changes such as switching to a vegetarian diet may be effective for prevention or management of LD-associated conditions such as non-alcoholic fatty liver disease and metabolic syndrome [106,107]. Diet-based interventions may represent a useful long-term strategy for controlling *Flaviviridae* infections, while SREBP pathway-based interventions are attractive for seasonal prophylaxis. Research into the use of SREBP pathway inhibitors such as PF-429242 should continue to outline the multifaceted effects of SREBP pathway manipulation in order to give insight about potential off-target, unwanted effects.

## Figures and Tables

**Table 1:** Summary of selected hepacivirus and flavivirus structural and non-structural (NS) proteins.

Virus Component	Hepatitis C Virus	Dengue and Zika Viruses
Genome	+ssRNA	+ssRNA
Capsid protein	Core *	C *
Envelope proteins	E1, E2	prM, E
Nonstructural proteins	NS2, NS3, NS4A, NS4B ^Δ^, NS5A *, NS5B	NS1, NS2A, NS2B ^Δ^, NS3 ^Δ^, NS4A ^Δ^, 2k, NS4B ^Δ^, NS5

* Indicates that the protein is LD-associated. ^Δ^ Indicates that the protein is not LD-associated but plays a proven role in SREBP pathway or lipophagy manipulation.

**Table 2:** Hepatitis C virus (HCV) and dengue virus (DENV) components involved in hijacking of the host sterol regulatory element-binding protein (SREBP) pathway for viral infection.

Virus	SREBP-Hijacking Action	Viral Factor (s)	References
HCV	Increases SREBP-1 and SREBP-2 expression in in vitro infection models	NS5A, NS4B, 3′ UTR	[45,46,47,50,51]
	Activates SREBP signaling in in vitro infection models	Core, NS4B, NS5A	[44,46,47]
	Reduces PCSK9, miR-24 and miR-223 concentrations in non-responders to antiviral treatment	Unknown	[31,42,52]
DENV	Hijacks host SKI-1/S1P to activate SREBP pathway	Unknown	[30]
	Cleaves host protein STING to prevent expression of interferon genes	NS2B, NS3	[53]

## Abbreviations

Ago2 AKT AMPK AUP1 Bax	argonaute 2 protein kinase B 5′ adenosine monophosphate-activated protein kinase ancient ubiquitous protein 1 Bcl-2 associated X protein
bHLH	basic helix-loop-helix
BVDV cLD	bovine viral diarrhea virus cytoplasmic lipid droplet
COPII COVID-19	coat protein II coronavirus disease-2019
CRISPR DAG	clustered regularly interspaced short palindromic repeats diacylglycerol
DAXX DDX3X DENV	death domain associated protein DEAD-box helicase 3 X-linked dengue virus
eLD	extracellular lipid droplet
ER	endoplasmic reticulum
HCV	hepatitis C virus
HSP IKKα IL-1β JEV INM INSIG	heat shock protein IκB kinase α interleukin-1β Japanese encephalitis virus inner nuclear membrane insulin-induced gene
LC3	microtubule-associated protein light chain 3
LD LDAF1 LDLR	lipid droplet LD assembly factor 1 low-density lipoprotein receptor
LVP MERS-CoV miR nLD	Lipoviroparticle Middle East respiratory syndrome coronavirus microRNA nuclear lipid droplet
NLS NS	nuclear localization signal non-structural
PCSK9 PI3K PML RNAi	proprotein convertase subtilisin kexin 9 phosphatidylinositol 3-kinase promyelocytic leukemia RNA interference
Raf1 S2P SARS-CoV	rapidly accelerated kinase 1 site-2 protease severe acute respiratory syndrome coronavirus
SCAP SKI-1/S1P SNARE	SREBP-cleavage activating protein subtilisin kexin isozyme-1/site-1 protease SNAP receptor
SRE	sterol regulatory element
SREBP STING	sterol regulatory element-binding protein stimulator of interferon genes
SVR S2P TAG TRIM16 UTR VLDL ZIKV 3D	sustained virologic response site-2 protease Triacylglycerol tri-partite-containing motif-16 untranslated region very low density lipoprotein Zika virus Three dimensional
